# Neuroprotective effects of *Morinda officinalis* How.: Anti-inflammatory and antioxidant roles in Alzheimer’s disease

**DOI:** 10.3389/fnagi.2022.963041

**Published:** 2022-09-08

**Authors:** Yi Zhang, Meng Zhang

**Affiliations:** Department of Gerontology and Geriatrics, Shengjing Hospital of China Medical University, Shenyang, China

**Keywords:** *Morinda officinalis* How., neuroprotection, neuroinflammation, antioxidative, Alzheimer’s disease

## Abstract

Pharmacological studies have shown that some traditional Chinese medicines (TCMs) have applications in the treatment of Alzheimer’s disease (AD). *Morinda officinalis* How. (MO) is a TCM with a long history and is widely used to tonify kidney Yang. *In vitro* and *in vivo* experiments have suggested that MO contains various effective pharmaceutical components and chemicals, including oligosaccharides, anthraquinones, iridoids, flavonoids, amino acids, and trace elements, conferring MO with anti-inflammatory and antioxidant properties. Neuroinflammation and oxidative stress are undoubtedly hallmarks of neurodegeneration, contributing to AD progression. In this mini-review, we summarize the molecular mechanisms, structure-activity relationships, and potential synergistic and antagonistic effects of active components in MO. This discussion highlights the roles of these active components, such as oligosaccharides, anthraquinones, and iridoid glycosides, in the treatment of AD *via* anti-inflammatory and antioxidant mechanisms, providing a scientific basis for further utilization of MO.

## Introduction

*Morinda officinalis* How. (MO) is a perennial vine of the genus *Rubidium coba* and is one of the “Four Major Southern Medicines” in China, together with areca nuts and *Alopecia solanum*. MO is mainly distributed in Fujian, Guangdong, Guangxi, and Hainan provinces in China (Yoshikawa et al., 1995; Wang et al., 2002). The main medicinal part of MO is its root, which is flat-cylindrical with a grayish-yellow surface and a bitter taste (Zhang et al., 2018). As a traditional Chinese medicine (TCM), MO belongs to the kidney and liver meridian and functions by tonifying kidney Yang, strengthening tendons and bones, and dispelling wind and dampness (Zhang et al., 2018). The “Shen Nong Ben Cao Jing” records state that, “MO can strengthen the body and bones, adjust the five-organ physiological function and enhance the immunity of the body.” Furthermore, as a TCM, MO warms the uterus, delays aging, and has antidepressant and antitumor effects.

The main components of MO include oligosaccharides, anthraquinones, iridoid glycosides, organic acids, trace elements, amino acids, and sterols (Zhang et al., 2022). Previous studies have suggested that oligosaccharides in MO can improve immunity, promote angiogenesis, enhance reproduction, and exert anti-osteoporosis, antioxidative, antidepressant, and antidementia effects (Xu et al., 2017; Jiang et al., 2018; Chi et al., 2020; Zhu et al., 2019). The iridoids in MO have antioxidant, anti-chromosomal mutagenesis, antitumor, anti-inflammatory, and analgesic functions (Choi et al., 2005; Zhang et al., 2020; Cai et al., 2021). Furthermore, palmitic acid and succinic acid present in MO have antidepressant effects (Cui et al., 1995; Zhu et al., 2020). Recent *in vivo* and *in vitro* experiments have confirmed that the bioactive components of MO easily cross the blood-brain barrier and play beneficial neuroactive roles in various aging and neurodegenerative disease models, including Alzheimer’s disease (AD) (Chen et al., 2017). This pharmacological mechanism of AD treatment may be realized through the anti-inflammatory and antioxidant mechanisms of MO active components (Wu Z. Q. et al., 2015; Luo et al., 2021). In the occurrence and development of AD, the overactivation of microglia and astrocyte, infiltration of T lymphocytes in brain tissue, peripheral macrophage entering brain tissue, decrease of normal phagocytosis function, increase of production and release of inflammatory cytokines, all of which can result in the aggravate of neuroinflammation and decrease of Aβ clearance (Hao Y. et al., 2020). Oxidative stress is another early change in patients with AD and leads to the production of reactive oxygen species (ROS) and free radicals, which are toxic to nerve cells (Chen and Zhong, 2014). ROS, as the main effector molecules in the process of oxidative stress, can lead to protein and lipid peroxidation, inhibit the activity of antioxidant enzymes, affect mitochondrial function, activate caspase-3, and induce neuron apoptosis through ASK1-JNK/p38 signal pathway, and involved in the pathogenesis of AD (Wu et al., 2020).

In this mini-review, we summarize the potential applications of MO in the treatment of AD and in anti-aging strategies *via* inhibition of neuroinflammation and antioxidant injury, aiming to provide a scientific basis for clinical use of MO in the treatment of neurodegenerative diseases.

## Botany and geographical distribution of *Morinda officinalis* How.

MO belongs to the family Rubiaceae, and there are approximately 102 species of *Morinda* worldwide, which are distributed in tropical, subtropical, and temperate regions, including China. Species of *Morinda* differ according to geographical location, and the medicinal parts of the plant also differ; the roots, leaves and fruits of MO all have medicinal properties (Wu Y. et al., 2015; Singh and Sharma, 2020). However, the main medicinal part is the roots (Choi et al., 2005). MO roots are mostly flat and cylindrical, are slightly curved, vary in length, and show a diameter of 0.5–2 cm. The surface of the root is a gray-yellow or dark gray, with longitudinal lines and transverse cracks, and some of the bark is broken off from the exposed wood (Yao et al., 2004; Huang et al., 2016). The texture of the root is tough, and the skin section is thick with a purple or lavender color. In 2014, researchers conducted field investigations on the plant resources of MO in Fujian, Hainan, Guangdong, and Guangxi and found that there were significant differences in the leaves and roots of MO from different habitats. Leaves of MO from Fujian are leathery and protuberant (Zhang et al., 2016), and plants can be divided into two categories based on the average leaf length. Some varieties of MO grow in clusters. The root flesh is compacted, and the shape varies. In Hainan MO, the leaves are leathery and smooth, and the root flesh is compacted and shows a rosary shape. By contrast, leaves of MO from Guangdong are leathery and protuberant, and the roots are fleshy and without obvious shape. The leaves of MO from Guangxi are leathery and smooth.

## Major phytochemical constituents in *Morinda officinalis* How.

MO contains carbohydrates, anthraquinones, iridoids, flavonoids, amino acids, and trace elements. Polysaccharides, one of the main active components of MO, have obvious biological activities, including antioxidant, antifatigue, immunoregulatory, and anti-osteoporosis effects, and may have diverse clinical applications (Zhang et al., 2018).

**FIGURE 1:**
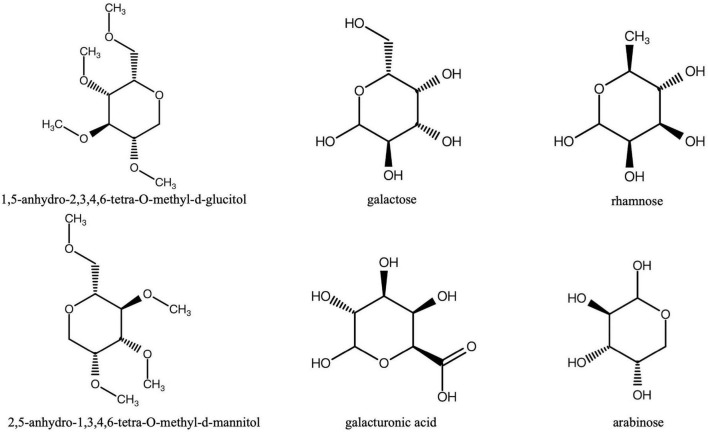
The structures of the main polysaccharides (MP-1, MP-2, MP-3) in *Morinda officinalis* How.

So far, a number of studies have reported that total or crude polysaccharides extracted from MO root have various biological activities, including the protective effect on bone loss (Liu et al., 2020), antioxidant (Zhang et al., 2013), anti-fatigue (Zhang et al., 2009) and immunomodulation (Choi et al., 2005), which suggests the polysaccharides of MO root exert important roles in its pharmacological properties (Zhang et al., 2009, 2013). Importantly, MO polysaccharides can scavenge free radicals and exert antioxidant effects (Soon and Tan, 2002). Three representative polysaccharides in MO, i.e., MP-1, MP-2, and MP-3, were found to have antifatigue activity in a weight-loaded swimming model in mice. The MP-1 were mainly identified as 1, 5-anhydro-2, 3, 4, 6-tetra-O-methyl-d-glucitol and 2, 5-anhydro-1, 3, 4, 6-tetra-O-methyl-d-mannitol; MP-2 consisted of arabinose, galacturonic acid, and galactose as the main constituents; MP-3 was an acidic polysaccharide with sulfur and nitrogen, and was rich in arabinose, as well as galacturonic acid and galactose (Figure 1; Zhang et al., 2009). In addition, MO-derived polysaccharides significantly inhibit the infiltration of neutrophils and macrophages into the liver, thereby acting as an immune regulator and alleviating hepatic injury (Xu et al., 2019). Inulin-type oligosaccharides extracted from MO (OMOs) can suppress behavioral changes in rats subjected to a single prolonged stress and cause an increase in the contents of all progesterone in the prefrontal cortex, hippocampus, and amygdala, suggesting that inulin-type oligosaccharides may have significant effects on alleviating symptoms of post-traumatic stress disorder (Qiu et al., 2016).

**FIGURE 2:**
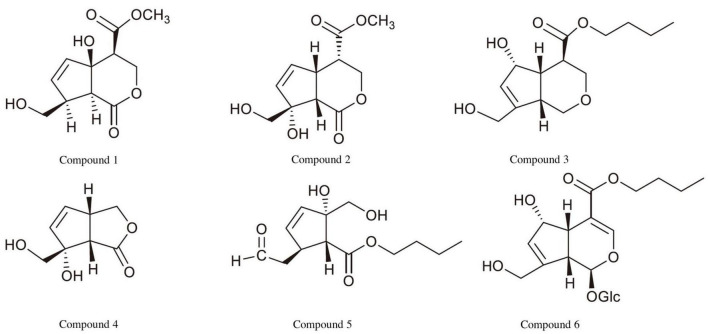
The structures of the main iridoids in *Morinda officinalis* How.

Iridoids are abundant and widely distributed in MO and are one of the main active components in MO (Hao Q. et al., 2020). Iridoids are mainly isolated from the roots, branches, leaves, and fruits of MO, and more than 50 iridoids have been isolated from MO to date (Cai et al., 2021; Figure 2). These MO-derived iridoids have various biological activities, including anti-inflammatory effects, blood flow enhancement, and antitumor effects. For example, in RAW264.7 macrophages, monotropein can inhibit lipopolysaccharide-dependent induction of tumor necrosis factor (*TNF*-α) and interleukin-1β (*IL-1*β) mRNA expression and reduce the activity of nuclear factor (NF)-κB (Shin et al., 2013). Furthermore, asperulosidic acid blocks polycoagulamine-induced erythrocyte aggregation and thrombin activity (Murata et al., 2014), suggesting that asperulosidic acid may have clinical applications in improving blood fluidity and could represent a novel therapeutic strategy for the prevention and treatment of thrombotic diseases. In terms of antitumor effects, citrifolinoside and citrifolinin A significantly inhibit the activity of ultraviolet-induced active protein 1, which plays an important role in tumor induction and growth (Sang et al., 2001; Tao et al., 2016).

Despite these findings, the effects of MO-derived compounds on the intestinal flora (Hu et al., 2016) and the potential therapeutic effects of these compounds in patients with AD have not been fully elucidated. Further studies are needed to explore the pharmacological activities of MO-derived compounds and elucidate the detailed mechanisms to provide a theoretical basis for the development of new drugs.

## Traditional Chinese medicines prescriptions containing *Morinda officinalis* How.

TCM prescriptions are usually composed of a variety of herbs in a certain proportion, and these herbs exert synergistic roles in the whole prescription, which is the basis for the TCM prescriptions to treat diseases through multi-target and multi-mechanism (Zhao et al., 2022). Several TCM prescriptions containing MO as the main ingredient have been shown to have anti-aging effects and efficacy against AD. Er-xian and Xiao-yao formulas contain primarily *Curculigo orchioides*, *Epimedium brevicornum*, and MO and have been shown to significantly reduce serum lipid peroxide (LPO) contents while enhancing superoxide dismutase (SOD) and catalase (CAT) activities in aging rats, suggesting potential anti-aging effects. Enhancement of antioxidant enzyme expression can increase the activity of antioxidant enzymes and suppress the generation of free radicals (Shen et al., 1995).

Bajitianwan (BJTW) is a TCM prescription with a long history and mainly consists of MO, *Acorus Tatarinowii* Schott, *Lycium Chinese* Mill, and *Poria Cocos* (Schw.) Wolf. BJTW has been used to treat cognitive impairment for hundreds of years. In a D-galactose-induced male Wistar rat aging model, BJTW significantly reduced the latency at which rats could find the target platform and increased the time of swimming in the target quadrant. BJTW also has positive effects on age-related memory impairment and may be a promising antioxidant candidate for the treatment of AD (Xu et al., 2020).

Dihuang Yinzi (DY) is a tonic prescription that nourishes kidney Yin, tonifies kidney Yang, opens the orificium, and resolves phlegm. MO is one of the major components of DY. DY can significantly alleviate the impairment of learning and memory in ischemic rats by increasing the expression of extracellular signal-regulated protein kinase and enhancing synapse formation (Hu et al., 2009). In traditional medicine, aging is believed to be closely associated with kidney deficiency. Because DY promotes kidney function, it may exhibit therapeutic effects in AD, in which aging is the main etiological factor (Lee et al., 2021). Moreover, DY has been shown to improve the cognition and energy metabolism of mice by protecting against mitochondrial injury (Zhu et al., 2014).

Huanshao Dan is mainly composed of yams, *Radix achyranthis bidentatae*, *Poria Alba*, and MO and has the effect of warming the kidney and toning the spleen. In a case-control study involving 309 elderly patients, Huanshao Dan significantly improved transient memory, reduced serum LPO levels, and enhanced SOD activity, resulting in clear kidney tonifying and anti-aging effects (Du et al., 1992).

Traditional prescriptions containing MO as the main ingredient have definite antidementia effects in both animals and aging patients (Kim et al., 2008). Early *in vivo* and *in vitro* studies also revealed that MO extracts may play neuroprotective roles through anti-inflammatory and antioxidant mechanisms, alleviating cognitive impairment in rat models of aging (Xie et al., 2001). Because MO extracts or active ingredients are mainly distributed in the intestinal tract, their effects on improving microbiota metabolism may also be one approach to alleviate cognitive impairment (Xie et al., 2004; Deng et al., 2015). The anti-inflammatory and antioxidant mechanisms of the main active components of MO in AD are further discussed below.

## Molecular mechanisms of action of *Morinda officinalis* How. in Alzheimer’s disease models

The neuroprotective effects of MO have been described in several AD and aging models, and the protective mechanisms involve mainly the anti-inflammatory and antioxidant effects of MO components (Table 1). For example, *bajijiasu* (BJJS), an oligosaccharide isolated from MO, tonifies the kidney and brain, resulting in significant improvement in the behaviors of rats with vascular dementia and enhancement of the long-term extension effects of synaptic transmission in isolated hippocampus slices in rats (Liang et al., 2005). BJJS also increases SOD and glutathione reductase contents and activities, decreases LPO contents, and reduces lipofuscin levels in brain tissues, thereby delaying the aging of brain tissue (Chen et al., 2014b). In APPswe/PSEN1ΔE9 (APP/PS1) double transgenic mice, Cai et al. (2017) demonstrated the BJJS inhibits neuroinflammation. They compared the levels of inflammatory factors between BJJS-treated and untreated APP/PS1 mice and found that NF-κB, IL-1β, and TNF-α expression, as well as the levels of the microglia markers Iba1 and CD40, were significantly decreased in the hippocampus and cortex of APP/PS1 mice treated with high-dose BJJS. Therefore, BJJS may improve cognitive performance in APP/PS1 mice by inhibiting the neuroinflammatory response. In addition to suppressing the neuroinflammatory response, high-dose BJJS significantly reduces Aβ1–42 and β-site app cleaving enzyme 1 levels in the hippocampus and cortex and increases the expression of neurotrophic factors, such as brain-derived neurotrophic factor (BDNF) and nerve growth factor (Cai et al., 2017).

**TABLE 1:** Pharmacological activities of *M. officinalis* How. in AD treatment.

Tested substance	Models	Tested living system/cell	Results	Dose range	Application time	Main anti-AD mechanism	References
*Bajijiasu*	APP/PS1 mice	Mice	Suppress the neuroinflammatory response, increase expression of neurotrophic factors	80 mg/kg/d	4 weeks	Inhibit neuroinflammation	Cai et al., 2017
OMO	D-Galactose/Aβ_25–35_ induced rat model	Rats	Alleviate oxidative damage, increase neurotransmitter levels and relative synaptophysin expression	480 mg/kg/d	4 weeks	Anti-oxidant effects	Deng et al., 2020
OMO	Aβ_25–35_ induced rats	Rats	Enhance oxidation resistance, activate brain energy metabolism and improve the injury of cholinergic system.	60 mg/kg/d	25 days	Enhance oxidation resistance	Chen et al., 2013b
*Bajijiasu*	Aβ_25–35_ induced cell	Pheochromocy toma cells	Reverse the reduction in cell viability, blockade of mitochondria-dependent apoptosis.	40 μM	2 h	Against oxidative stress	Chen et al., 2013a
*Bajijiasu*	APP/PS1 mice	Mice	Reduced ROS and MDA levels, and alleviate endoplasmic reticulum stress.	70 mg/kg/d	4 weeks	Alleviate oxidative stress	Xu et al., 2018
*Bajijiasu*	Aβ_25–35_ induced rats	Rats	Enhance antioxidative activity and energy metabolism, and attenuate cholinergic system damage	2 g/kg/d	2 weeks	Inhibit oxidative stress	Chen et al., 2014b
OMO	APP/PS1 transgenic/C57BL/6J male mice	Mice	Improve in the gut microbiome and metabolome	100 mg/kg/d	4 weeks	Regulate the key microbiota-metabolite pairs	Xin et al., 2019
FOS	D-Galactose/Aβ_25–35_ induced rat model	Rats	Alter the gut structure of the microbiota, promote the engraftment ability of Bifidobacterium	100 mg/kg/d	28 days	Alter the diversity and stability of the microbial community	Chen et al., 2017

AD, Alzheimer’s disease; OMO, oligosaccharides of Morinda officinalis How.; FOS, fructose-oligosaccharides.

A recent study revealed that OMOs can significantly enhance learning and memory abilities and alleviate symptoms of dementia (Chen et al., 2013b). Deng et al. (2020) further explored the pharmacological effects of these oligosaccharides in a D-galactose- and Aβ 25–35-induced rat model of AD and found that OMOs exerted neuroprotective effects *via* antioxidant-related mechanisms in the hippocampus and cortex of AD rats. Furthermore, in a rat model of AD, OMOs were shown to alleviate oxidative damage in the brain, and oligosaccharides significantly increased in the activities of CAT, SOD, and glutathione peroxidase in the hippocampus, while decreasing the content of malondialdehyde. In addition to protecting brain tissues from oxidative damage, OMOs may protect against D-galactose- and Aβ25–35-induced AD by increasing neurotransmitter levels and relative synaptophysin expression levels in AD model rats; thus, OMOs may represent a novel strategy for alleviating symptoms of AD in the clinical setting (Deng et al., 2020).

BJJS has also been shown to protect against Aβ25–35-induced neurotoxicity in pheochromocytoma cells and rats (Chen et al., 2013a). To further explore the neuroprotective mechanisms of BJJS, Chen et al. (2014b) evaluated whether BJJS inhibited oxidative stress and neural apoptosis. In an Aβ25–35-induced model of learning and memory dysfunction in rats, BJJS administration promoted SOD production, significantly increased CAT and glutathione peroxidase levels, and inhibited malondialdehyde production. Therefore, enhancement of antioxidative activity may be the main mechanism of BJJS neuroprotection. In addition, BJJS was shown to promote energy metabolism in the brain tissue of AD model rats and increase the level of acetylcholine (ACh), suggesting another potential therapeutic mechanism for BJJS efficacy in patients with AD (Chen et al., 2014b). In the APP/PS1 mouse model, BJJS also showed potential applications in the treatment of AD. Indeed, in a study by Xu et al. (2018) BJJS was shown to exert AD therapeutic effects by altering the oxidative stress reaction. In addition, BJJS can significantly reduce the levels of lipid peroxidation and ROS in APP/PS1 mice, promotes the expression of BDNF, and inhibits the endoplasmic reticulum stress response, thus indicating the neuroprotective effects of BJJS on cognitive dysfunction in APP/PS1 mice were associated with protection against oxidative stress, apoptosis, and endoplasmic reticulum stress (Xu et al., 2018).

The role of the gut microbiota in neurodegeneration is attracting much interest (Ho and Ross, 2017), and the development of new therapeutic strategies for AD may be facilitated by studies of the pathogenic role of the gut microbiota in AD (Pistollato et al., 2016). Clinically applied sodium oligomannate capsules may show efficacy in the treatment of AD by affecting the gut microbiota (Wang T. et al., 2020). Additionally, OMOs have been reported to delay the progression of AD in APP/PS1 transgenic mice and C57BL/6J male mice by regulating the diversity of the intestinal microbiota and metabolic components (Xin et al., 2019). In previous studies, *Bacteroidetes* levels were decreased, whereas *Firmicutes* levels were increased in APP/PS1 transgenic mice (Wang et al., 2015; Dao et al., 2016), and OMOs were shown to reverse these changes. In addition, through modulation of the intestinal microbiota, OMOs may regulate protein expression and intracellular signaling pathways and significantly reduce the levels of Aβ1–42 in the brain tissues of AD mice. Moreover, OMOs affect the number of typical gastrointestinal tract microorganisms, such as *Lactobacillus*, *Bifidobacterium*, and *Bacteroides*, in APP/PS1 transgenic mice; alter intestinal morphology, mucin production, and intestinal permeability; and reduce bacterial imbalances (Xin et al., 2018). Fructose-oligosaccharides (FOSs) are generally considered prebiotics because they stimulate the growth of *Bifidobacterium* and *Lactobacillus*. A previous study showed that FOSs derived from MO effectively improve memory in AD model animals; however, the specific neuroprotective mechanisms remain unclear (Chen et al., 2014a). In a rat model of AD, Chen et al. (2017) studied the therapeutic effects of FOSs on AD *via* multiple pathways. First, FOSs were found to enhance antioxidant activity and alleviate D-galactose-induced learning and memory dysfunction in rats. Second, ACh levels were shown to be significantly increased in AD model rats treated with FOSs, and FOSs alleviated brain swelling and neuronal apoptosis and decreased the expression of intracellular markers (Tau and Aβ1–42). Third, FOSs were found to alter the diversity and stability of the microbial community, enhance the inflammatory environment, promote the secretion of certain monoamine neurotransmitters (e.g., NE, DA, and 5-HT) in a concentration-dependent manner, and alleviate the learning and memory dysfunction induced by Aβ1–42 in rats (Chen et al., 2017).

In summary, MO and its active components exert anti-AD effects mainly through antioxidant and anti-inflammatory mechanisms. Additionally, MO extract may also relieve AD symptoms through other mechanisms, including inhibition of acetylcholinesterase, butyrylcholinesterase, beta-site amyloid precursor protein cleaving enzyme 1, and advanced glycation end-products. These effects of MO extracts are exerted *via* anthraquinones, coumarin, and phytosterol, which have been isolated using a bioassay-guided approach; therefore, MO root extracts may have therapeutic effects as a natural medicine for treating AD (Lee et al., 2017).

## Perspectives and conclusion

Despite extensive research on the pathogenesis of AD, no single theory can fully explain the pathogenesis of AD, which involves multiple inter-related molecular signaling pathways (Chakravorty et al., 2019; Agrawal and Jha, 2020). Moreover, it is difficult for single-component drugs to achieve satisfactory therapeutic effects against a certain target. As described above, various TCM prescriptions containing MO as the main ingredient have neuroprotective and antidementia properties. Studies have shown that these Chinese herbal prescriptions may show efficacy against AD by inhibiting oxidative stress, neuroinflammatory injury, apoptosis, and other biological processes (Hao et al., 2019; Li et al., 2003). Indeed, TCM prescriptions are typically advantageous because they contain multiple components and target multiple mechanisms and pathways (Shen et al., 2019; Wang K. X. et al., 2020). One of the difficulties in the study of TCM prescriptions is the complexity of the medicine. Therefore, many studies have focused on identifying and characterizing the monomers and active components of TCMs.

**FIGURE 3:**
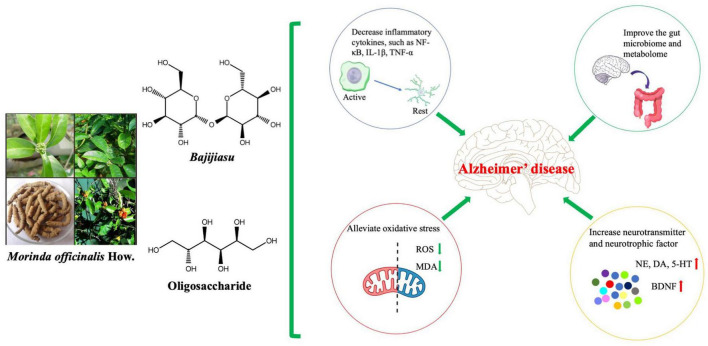
Antioxidative and Anti-inflammatory activities of *Morinda officinalis* How. (MO) and its main components. MO may be used to treat Alzheimer’s disease and improve functional behavioral outcomes by reducing oxidative stress, inhibiting inflammatory factors, enhancing the intestinal microbiome and metabolome, and increasing neurotransmitter and neurotrophic factor expression. 5-HT, 5-hydroxytryptamine; BDNF, brain-derived neurotrophic factor; DA, dopamine; IL-1β, interleukin-1β; MDA, malondialdehyde; NE, norepinephrine; NF-κB, nuclear factor kappa-B; ROS, reactive oxygen species; TNF-α, tumor necrosis factor α.

Among the active components of MO, BJJS and OMOs have been the most extensively studied with regard to the treatment of AD using *in vivo* and *in vitro* experiments (Figure 3). Other active components of MO, such as iridoid glycosides, have significant anti-inflammatory effects (Zhang et al., 2020), and pharmacokinetic studies have suggested that iridoid glycosides are mainly distributed in the intestinal tract, suggesting potential AD therapeutic effects *via* alteration of the intestinal inflammatory response (Gao et al., 2021). However, existing studies still have some limitations. First, in terms of chemical composition, extensive screening of drug components has been carried out, and targeted studies should be performed based on the pharmacological action of MO. Furthermore, studies of the pharmacological effects of MO, the modeling methods and observation indexes were relatively simple, and many experiments were reproducible. Finally, in terms of pharmacological studies, most existing studies have focused on the observation and evaluation of drug efficacy, without in-depth and systematic exploration of the mechanisms of actions.

MO has been widely used in TCM prescriptions, and the biological effects of its active components have been confirmed in many studies. The findings of these studies have suggested that MO may have multipotent neuroprotective functions elicited through its anti-inflammatory and antioxidant effects. Accordingly, MO may have applications in the treatment of aging and AD. However, the synergistic effect, safety, efficacy, bioavailability, and metabolism of the components of MO need to be further studied.

## Author contributions

YZ and MZ wrote the manuscript. YZ produced the figures. MZ contributed to the editing and revision of the review. Both authors have read and approved the final manuscript.
